# Supplementary material for: Are carbon emissions trading and green financial instruments synergistic? -Comprehensive quantitative research based on content analysis
Source: PLoS One. 2024 Mar 7;19(3):e0298601. doi: 10.1371/journal.pone.0298601 (PMC10919601; doi:10.1371/journal.pone.0298601)
Supplement: S1 Data — (ZIP) [file pone.0298601.s001.zip › Data/地级市Z_F工作报告.zip!/╔┬╬≈/═¡┤¿╩╨/└┤╫╘╣1⁄2╓┌║┼í╛┬φ┐╦╩2╛▌═°í┐_2008.docx]

各位代表：

　　现在，我代表市人民政府向大会作工作报告，请予审议，并请市政协委员和其他列席人员提出意见。

　　一、2007年工作回顾

　　过去的一年，在市委的坚强领导下，各级政府和全市人民认真贯彻党的十七大精神，以邓小平理论和“三个代表”重要思想为指导，全面落实科学发展观，按照市第十次党代会的部署，大力实施“5523”工程，团结奋进，锐意进取，圆满完成了市十四届人大二次会议确定的目标任务，全市经济社会发展呈现出速度加快、结构优化、效益提高、民生改善的良好态势。

　　（一）经济发展增势强劲。全年实现生产总值102.27亿元，增长15.3%，增幅居全省第三位，是1984年以来增长最快的一年。财政总收入12.09亿元，增长42.5%，其中地方财政收入6亿元，增长47.6%，增幅均居全省第二位。规模以上工业完成总产值122.14亿元，增长25.9%；实现利润6.92亿元，增长63.9%；实现税金7.04亿元，增长27.4%；工业经济效益综合指数154.8，较上年提高了25.6个百分点。电解铝产量17万吨，煤炭1749万吨，水泥695万吨，水泥熟料599万吨，分别增长31.3%、3.6%、22.3%和17.7%。工业对经济增长的贡献率达到63.3%。三次产业结构为7.7：57.5：34.8。

　　（二）项目建设成效显著。全社会固定资产投资完成57.03亿元，增长32.5%。“十一五”重大项目建设完成投资41.8亿元。华能电厂一期工程提前三个月实现双投。铜川铝业技改续建、玉华煤矿改扩建、西川煤矿、秦岭200万吨水泥粉磨站、中星耐磨复合管、30万吨水煤浆、330千伏金锁变增容改造等11个项目建成投产或运营。铜川矿区采煤沉陷区综合治理、玉皇阁大桥、南市区基础设施建设等项目进展顺利。声威第二条日产4500吨水泥熟料生产线、旺仔小馒头等项目开工建设。

　　（三）新农村建设稳步推进。农业综合生产能力进一步提高。粮食总产26.7万吨，增长7.4%。新栽水果5.7万亩、干杂果11.3万亩，苹果总产32.5万吨，增长8.9%，优果率72%。10个奶牛养殖示范小区基本建成，新增奶牛3229头。启动实施“一村一品”示范村105个、扶贫开发重点村71个。农村信息化建设通过省上验收。建成通村公路327公里、通乡油路87.5公里，解决了5.6万农村人口饮水安全问题，新增沼气用户6150户，移民搬迁7060人。全年植树造林17万亩，治理水土流失153.7平方公里。

　　（四）节能减排力度加大。关闭拆除了8台水泥机立窑和1台中空回转窑生产线，淘汰落后水泥产能75万吨。加大了水泥、电解铝等行业节能改造力度，吨水泥熟料能耗下降13千克标煤，吨铝电耗下降118千瓦时。万元生产总值能耗下降4.7%，二氧化硫排放量、化学需氧量分别削减3.88%和2.4%。城市空气质量二级以上天数达到296天，较上年增加20天。

　　（五）第三产业发展较快。编制了全市旅游发展总体规划。药王山南旅游环线建成通车，陈炉古镇景区开发、照金红色旅游、耀州窑金元遗址保护等项目开工建设。举办了玉华冰雪节、新区牡丹节等活动，接待国内外游客346万人次，实现旅游综合收入4.2亿元，分别增长8.1%和21.4%。居民消费价格指数上升4.7%，社会消费品零售总额29.82亿元，增长17.5%。

　　（六）创卫工作取得阶段性成效。完成了宜园路、金华路、永安北路等城市道路改造和正阳路、长虹路等非机动车道、人行道建设及绿化亮化工程，加宽改造了七一桥、红旗桥，铺设更新人行道14万平方米。漆水河河滨景观综合改造基本完成。建成垃圾中转站12座，新建改造城市公厕93座，城中村、卫生村户厕改造8659户。新耀污水处理和垃圾无害化处理工程进展顺利。新增城市绿地110万平方米，绿化覆盖率达到37.6%。210国道沿线环境综合整治初见成效。创卫的8项基本条件已有6项基本达标，城市面貌和市民素质有了较大提升。

　　（七）改革开放继续深化。28户国有企业改制任务基本完成。5户困难企业政策性破产通过国家批准，启动了西北耐火材料厂、市制药厂、市啤酒厂政策性破产程序，打包核销国企债务3.05亿元。秦岭水泥公司通过资产重组实现了扭亏保壳。财税、投资和国有粮食企业改革有序推进。新登记私营企业381户、个体工商户1560户。全年招商引资签订合同项目131个，有100个项目开工建设，实际到位资金11.5亿元。外贸进出口总额2300万美元，增长25%，其中出口1110万美元，增长73%。

　　（八）社会事业取得新成绩。推广转化科技成果10项，新增民营科技企业6家。“两基”工作通过国家评估验收。完成农村中小学危房改造4.5万平方米，30所省属企业学校和8所市直学校划归区县管理。新型农村合作医疗农民参合率达到94%，人均补助标准居全省第一位。新区医院一期工程投入使用。建成标准化村卫生室131个、社区卫生服务机构31个。实施社区服务设施建设项目16个。建成乡镇文化站4个、村文化室10个。铜川体育馆建成使用。完成铜川电视转播台技术改造，新增数字电视用户3500户。全市人口自增率4.44‰。深入开展了“五五普法”和依法治理工作，创建民主法制示范村187个，完成了29个基层司法所建设任务。安全生产四项指标全面下降，全市社会大局稳定。

　　（九）人民群众得到更多实惠。全年新增就业1.35万人，城镇登记失业率4.4%，劳务输出6.65万人次，基本消除了城镇零就业家庭。7.45万城镇人口和1.17万农村人口纳入低保范围，提高了90岁以上高龄老人的生活保健补贴标准，为7500户困难居民发放了取暖补贴，500多名贫困大学生得到资助。香港“健康快车”为我市1138名贫困白内障患者免费实施了复明手术。新开工住房30.2万平方米，建成23.1万平方米，其中廉租房5.7万平方米，安置困难群众2172户。城镇居民人均可支配收入8163元，增长19.7%；农民人均纯收入2620元，增长18.3%，增幅均居全省第二位，增加额是改革开放以来最多的一年。市委、市政府为全市人民办理的20件实事全面完成。

　　（十）政府自身建设不断加强。公共突发事件应急机制初步建立。依法行政和政务公开工作扎实推进，市级电子政务平台建设走在全省前列。市政府定期向市人大常委会报告工作，不断加强与市政协的联系和沟通，全年办理市人大代表建议67件、政协提案196件。廉政建设和反腐败斗争深入开展，部门和行业风气继续好转。

　　审计、统计、物价、人防、供销、气象、地震、档案、外事、民族、宗教等方面的工作都取得了新的成绩！

　　各位代表，2007年是我市发展进程中非常重要的一年，各项工作取得了令人鼓舞的成绩。这是市委驾驭全局、正确领导的结果，是市人大、市政协和各民主党派及社会各界监督支持的结果，是全市人民同心同德、奋发进取的结果。我代表市人民政府，向奋战在各条战线上的工人、农民、知识分子、干部、驻铜部队指战员、武警官兵和公安干警致以崇高的敬意！向大力支持政府工作的各位代表、各位委员和社会各界人士表示衷心的感谢！

　　在发展形势较好的情况下，更要清醒地认识到，全市经济社会发展还存在不少困难和问题。经济结构不尽合理，第三产业和非公有制经济发展不快，国企改革滞后；经济发展方式较为粗放，节能减排形势严峻；接续建设的大项目、好项目较少，投资快速增长的基础还不牢固；城乡居民收入在全省仍处于较低水平，一些涉及群众切身利益的问题还比较突出，维护稳定的压力较大；机关作风亟待改进，干部的精神状态、创新能力与新形势、新任务的要求还有许多不相适应的地方。对此，必须采取更加有力的措施，切实加以解决。

　　二、2008年经济社会发展的主要目标任务

　　今年是全面贯彻落实党的十七大精神、实现率先发展跨越发展的关键一年，我们将迎来举世瞩目的北京奥运会、改革开放30周年和铜川建市50周年，做好今年的工作具有十分重要的意义。目前，铜川经济社会发展正处在上升阶段，近年来项目建设积蓄的势能正在逐步释放，经济发展的内生动力不断增强，广大群众富民强市的愿望更加迫切，为我市加快发展提供了强大的思想基础、物质条件和动力源泉。同时，还应该看到，当前国际国内形势出现不少新的复杂因素。世界经济增长放缓，国际市场石油、粮食价格高企和美国次贷危机带来的风险加大。我国接连发生历史罕见的低温雨雪冰冻灾害、四川汶川特大地震灾害和南方水灾等一系列自然灾害，使灾区人民生命财产遭受严重损失，也给我市经济社会发展造成一定影响。中央实施“两防一从紧”政策，从严控制新增建设用地，严格控制新开工项目，经济发展的宏观约束日益趋紧。加之物价指数居高不下，保障煤电油运供应难度加大，经济运行的不确定因素和潜在风险明显增多。我市经济社会发展面临着前所未有的挑战和考验。我们要按照国家和省上的决策部署，一手坚持不懈地抓抗震救灾工作，一手坚定不移地抓经济社会发展，积极主动地做好风险防范工作，不断开创经济社会发展的新局面。

　　今年全市经济社会发展的总体要求是：以党的十七大精神为指导，深入贯彻落实中央经济工作会议、省委十一届二次全会和市第十次党代会、市委十届三次全会精神，围绕建设以现代建材业为主导的新型工业城市、实现率先发展跨越发展的目标，坚持“5523”发展思路，大力实施项目带动、工业强市和可持续发展战略，着力优化经济结构和提高经济发展质量，积极推进改革开放和自主创新，加强节能减排和生态环境保护，加快新农村建设，提升城市形象，全力实施“民生十大工程”，促进社会和谐，推动全市经济社会又好又快发展。

按照“既要积极进取，又要切实可行，并适当留有余地”的要求，今年经济社会发展的主要预期目标是：生产总值增长13%，达到120亿元；全社会固定资产投资完成50亿元；财政总收入同口径增长26.5%，达到14.64亿元；地方财政收入同口径增长28.4%，达到7.2亿元；剔除华能电厂后，万元生产总值能耗下降4%；二氧化硫排放削减4%、控制在1.85万吨以下，化学需氧量排放削减2.8%、控制在0.82万吨以下；城镇新增就业1.2万人，城镇登记失业率控制在4.5%；城镇居民人均可支配收入增长13%、达到9224元，农民人均纯收入增长12%、达到2934元；人口自增率控制在5.5‰以内。

　　实现今年经济社会发展的目标任务，要着力把握好以下五项原则：

　　一是坚持项目带动，实现又好又快发展。项目是铜川经济社会发展的生命线。要坚定不移地实施项目带动战略，坚持工作项目化、机遇项目化、优势项目化，使第一要务体现在抓项目、跑招商、引资金、聚产业上。大力优化经济结构，切实转变发展方式，在资源上做文章、在区位上挖潜力、在创新上找出路、在循环经济上增效益，实现经济总量迅速扩张和经济效益显著提升。

　　二是坚持改革开放，推进机制创新。充分发挥市场配置资源的基础性作用，加快以市场为取向的各项改革步伐，充分利用国际国内“两个市场、两种资源”，整合一切可以整合的要素，利用一切可以利用的资源，调动一切可以调动的力量，努力构筑充满活力、富有效率、更加开放、有利于科学发展的体制机制。

　　三是坚持统筹发展，加快城乡一体化进程。要把新农村建设作为促进城乡一体化的关键之举。加强农业基础建设，促进农业发展、农民增收。建立以工促农、以城带乡长效机制，促进公共服务均等化，努力形成城乡经济社会发展一体化新格局。

　　四是坚持以人为本，促进社会和谐。加快推进以改善民生为重点的社会建设，更加重视解决关系群众切身利益的突出问题。千方百计扩大就业，增加城乡群众收入，加快发展社会事业，高度重视安全生产，努力维护社会稳定，共建共享和谐社会。

　　五是坚持永续发展，建设生态文明。把节能减排作为促进科学发展的重要抓手和检验发展成效的重要标准。节约能源资源，保护生态环境，集约高效用地，使节能环保成为政府决策的基本要求、企业发展的硬性约束和全社会的自觉行动，努力建设资源节约型、环境友好型社会。

　　各位代表，今年时间即将过半，从目前全市经济社会发展情况看，上半年有望实现时间任务“双过半”。但和全国、全省一样，我市经济社会发展遇到了前所未有的挑战，我们一定要增强责任意识和忧患意识，弘扬伟大的抗震救灾精神，以百倍的努力做好下半年的各项工作，努力保持全市经济社会又好又快发展的良好势头，确保今年各项目标任务的全面完成。

　　三、以项目建设为支撑，筑牢又好又快发展的基础

　　今年计划实施重点建设项目55个，完成投资39亿元，其中建成项目31个。举全市之力，着力抓好20个投资过亿元的重大项目，努力在大项目、好项目建设上实现新突破。

　　大力推进产业项目建设。加快声威、秦岭、耀州、满意、凤凰等日产4500吨水泥熟料生产线建设步伐。开工建设年产12万吨碳素、10万吨铝板、3万吨铝合金棒生产线。加快煤炭资源整合和煤矿扩能技改步伐，开工建设白石崖煤矿改扩建项目，打造照金、西川、柴家沟、白石崖、秀房沟等地方主力矿井。加快旺仔牛奶、佰度食品、中星防腐耐磨管材等项目建设，促进产业结构优化升级。

　　加快基础设施项目建设。建成玉皇阁大桥及其连接线工程，全力做好西铜高速公路改扩建工程环境保障工作，开工建设后烈桥至店头二级公路。完成采煤沉陷区治理工程，基本建成裕丰园、梅苑、玉华等5个住宅小区，累计搬迁安置群众1万户，治理改造学校、幼儿园50所。实施35千伏陈炉变升压改造工程，建成110千伏关庄变增容改造工程。城镇居民用电一户一表改造1万户。抓紧修复因地震影响受损的基础设施，确保供水、供电、供气、通讯等安全运行。

　　抓好项目储备和落实工作。加大项目开发力度，使达到可研程度的项目滚动保持在100个以上。公布实施“十一五”第三批重大项目。安排项目前期费用和产业化发展资金500万元，精心策划、包装、推介一批重大项目。对列入计划的重点前期项目，实行一个项目一套班子，全程跟踪负责。华能电厂二期、铜川铝业自备电厂增容改造项目完成可研报告并力争报批，争取龙潭水库开工建设。继续坚持包抓领导、责任部门、项目法人“三位一体”的项目包联责任制，加强环境保障，搞好协调服务，确保项目顺利实施。

　　四、以园区为载体，做大做强现代建材业

　　要着力转变工业发展方式，壮大优势产业，培育新兴产业，加速生产要素聚集，大力推进新型工业化进程。

　　壮大优势特色产业。按照大区域谋划、大产业构建、大集团引领、大项目支撑的思路，提升壮大传统优势产业，培育发展新兴产业。加大对铝业、水泥、陶瓷等优势产业的支持力度。加快煤炭就地转化，确保华能电厂满负荷运转。大力发展水泥及水泥制品、墙体材料，提高附加值。坚持政府引导、整合资源、市场运作、园区启动，在引进实施重大陶瓷产业项目上取得实质性进展。抓住大飞机制造落户阎良和陕汽集团等装备制造业扩张的机遇，以东昌车桥公司、陕西管件设备厂、铜川煤矿机械厂为依托，搞好产业对接，实施一批零配件加工、装备制造业项目。积极发展食品、医药、电源、新材料等新兴产业，不断提高对经济增长的贡献率。

　　大力推动园区化发展。按照产业发展集群化、集群发展园区化的思路，完善市级六大工业园区规划。加强基础设施和产业配套环境建设，提高园区产业承载能力。充分发挥铜川铝业、秦岭、声威、中星等龙头企业的辐射带动作用，引导同类企业和相关项目向园区聚集。坚持品牌带动、质量兴市，推进技术创新，提高产品科技含量，努力把秦岭、声威、中星培育成中国驰名商标。

　　加强工业运行协调。坚持经济运行月通报、季分析制度，增强调控的主动性和前瞻性。突出抓好重点行业、骨干企业和主要产品的跟踪分析，做好煤电油运的保障工作，协调解决好华能电厂电煤供应、铜川铝业电价等问题。确保全市规模以上工业产值增长25%，达到150亿元。

　　五、以新农村建设为统领，促进农业发展农民增收

　　把解决好“三农”问题作为重中之重，认真贯彻中央1号文件精神，不折不扣地落实各项强农惠农政策，加强农业基础建设，发展农业主导产业，增强农村经济发展活力。

　　提升主导产业发展水平。粮田面积稳定在105万亩，粮食总产达到20万吨以上。大力推进“一村一品”工程，抓好示范村建设，促进果牧主导产业扩大规模、提质增效。新栽水果5万亩、干杂果6万亩，全面建成160万亩鲜干果基地。苹果套袋16亿只，总产34万吨，优果率达到73%。新增“一村一品”水果专业村10个、干杂果专业村10个，建成市级标准化水果示范园3万亩。大力推广奶牛标准化饲养，新增奶牛3000头。认真落实扶持生猪生产的各项政策，扩大生猪养殖规模。以无公害设施蔬菜为重点，统筹发展精细菜和大路菜，蔬菜产量达到10万吨以上。因地制宜发展中药材、烟叶种植。积极发展各类专业协会和专业合作经济组织，实现农户分散经营与市场的有效对接。

　　加强农业基础建设。整合支农资金，捆绑使用，集中投放，提高资金使用效益。启动新农村建设重点示范村16个、扶贫重点村20个，移民搬迁5000人。铺设县乡油路90公里、通村公路230公里，建成4个乡镇汽车站。解决3万人的饮水安全问题，新增沼气用户4000户，建设农村信息服务站300个。新修基本农田1.3万亩，发展节水灌溉2万亩。确保耕地保有量124万亩，基本农田保护面积118万亩。加强人工增雨和防雹体系建设，提高农业抗御自然灾害的能力。

　　发展壮大县域经济。加快工业强县步伐，市财政对区县工业园区基础设施建设给予适当补助，搭建县域工业发展平台，扶持农产品加工业、特色优势产业、劳动密集型产业和大工业配套产业发展。加快中小企业信用评估和担保体系建设，破解县域经济发展的资金制约。合理划分市县两级的事权财权，落实属地统计制度，强化县域经济领导责任，充分调动区县的积极性，力争县域生产总值平均规模达到26亿元。

　　深化农村综合改革。稳定和完善农村基本经营制度，完成农村综合改革年度任务。继续推进乡镇机构改革，完善乡财乡用县监管、村财村用乡代管制度。全面完成畜牧兽医管理体制改革，做好种子企业和农技推广体系改革工作，完成耀州区集体林权制度改革试点任务。

　　六、以现代服务业为重点，大力发展第三产业

　　加快发展第三产业是优化产业结构、转变发展方式、实现节能减排目标的迫切需要。创新发展方式，加大扶持力度，努力在发展传统服务业、培育现代服务业上实现新突破，力争第三产业增加值占生产总值的比重提高2—3个百分点。

　　加大第三产业扶持力度。认真贯彻《国务院关于加快发展服务业的若干意见》，编制第三产业发展规划，将第三产业中、长期目标逐年量化，层层分解下达，实行年度考核。设立第三产业扶持专项资金，支持现代服务业中的优势产业、新兴领域和薄弱环节。鼓励和引导非公有制经济发展第三产业，通过兼并、联合、重组等方式，促进规模化、品牌化、网络化经营。

　　加快发展旅游产业。加强旅游基础设施建设，改善景区环境。玉华宫争创4A级旅游景区，启动药王山大景区建设，完成照金红色旅游项目建设任务，编制香山景区开发规划。做好耀州窑、陈炉陶瓷古镇的保护与开发，力争耀瓷产品地域性保护通过国家批准。办好玉华冰雪节等节庆活动。全年接待国内外游客367万人次，实现旅游综合收入4.66亿元。

　　提高服务业发展水平。积极引进战略投资者，改造提升商贸流通业，大力发展连锁经营、特许经营等现代经营方式和新型业态，满足群众多层次、多样化的消费需求。优先发展运输业，提升物流的专业化、社会化服务水平。大力扶持新区服务业发展，启动专业街区建设，完善商业服务设施，改善南市区商务环境。落实鼓励消费的政策措施，提高居民的消费预期，扩大即期消费规模。全年实现社会消费品零售总额34.5亿元，增长16%。

　　转变外贸发展方式。提高对现有外贸企业的服务水平，加大外贸经营人员培养力度，加强与西安海关等部门的联系合作，扩大医药、电池、果汁、服装等主导产品出口，促进对外贸易快速发展。全年进出口总额达到2800万美元，增长21.7%。

　　加强农村市场体系建设。搞好农村集贸市场规划和建设，大力发展农村连锁超市，新建标准化农家店50个。支持连锁企业向农村延伸，促进农村消费升温。完善农产品流通体系，发展各类流通中介组织，培育涉农商贸企业，促进农产品销售。

　　七、以节能减排为切入点，着力建设生态文明

　　牢固树立节约优先、环保优先的理念，努力形成节约能源资源和保护生态环境的产业结构、发展方式、消费模式。

　　打好节能减排攻坚战。随着华能电厂、铜川铝业技改续建等项目的建成投产，能耗净增较多，实现节能目标压力很大。要全面落实节能减排综合性工作方案，健全统计监测考核体系，加强对27户重点用能单位的监管，加速淘汰水泥、冶炼、电石等行业的落后产能。全年淘汰落后水泥产能100万吨。提高项目环境准入门槛，认真执行环境影响评价制度，加强对新上项目的节能评估审查和污染物排放总量审批，从严控制高耗能、高污染、浪费资源的建设项目。大力发展循环经济，加快实施声威、秦岭窑尾余热利用等重点节能工程，促进煤层气、煤矸石、粉煤灰的综合开发，实现资源的高效节约利用。加强建筑节能管理，城镇新建建筑全面执行节能设计标准，新型墙材应用达到96%以上。做好机关节能工作，人均资源消耗下降5%。

　　加大环境污染治理力度。完成全市第一次污染源普查工作。加大对210国道沿线水泥企业的监管力度，重点抓好药王山周边水泥企业的污染治理，关闭淘汰5条湿法旋窑生产线。深化市区煤烟污染治理，积极推广水煤浆等清洁能源，改造燃煤锅炉20台，新增天然气用户3000户，确保城市空气质量好于二级以上天数达到266天。完善北市区污水收集管网，加快建设新耀污水处理及回用工程，不断提高污水处理率和中水利用率。加强城市饮用水源保护，推行水源区水质月报制度。

　　大力推进生态建设。广泛开展全民植树造林活动，认真抓好“三化一片林”绿色家园、天然林保护、退耕还林、“三北”防护林等林业重点工程建设，210国道直观山坡重点区域造林2000亩，完成二道桥废弃矿山立体绿化和三里洞矿东山坡绿化。全年植树造林15万亩，治理水土流失70平方公里。通过盘活关闭破产企业土地、城中村改造、耕地占补平衡等方式，充分挖掘存量土地潜力。加大“空心村”整治力度，搞好土地开发整理。整顿矿产资源开发秩序，建立合理的生态补偿机制。

　　各位代表，节约资源、保护环境，功在当代、利在千秋。我们要本着对未来发展、对子孙后代高度负责的精神，持之以恒地抓好资源节约和环境保护，努力使人民群众喝上干净的水、呼吸上清洁的空气、吃上放心的食物，在良好的环境中生产生活！

　　八、以增收节支为主线，提高公共财政保障能力

　　深化预算制度改革，强化预算约束，优化财政收支结构，为改革发展稳定提供强有力的财力保障。

　　促进财政增收。把开源的重点放在工业上，把挖潜的重点放在第三产业上，放水养鱼，支持企业的正常经营和发展，做强骨干财源，做大后续财源。要依法治税，依率计征，做到应收尽收，努力提高财政收入在生产总值中的比重。启动非税收入管理改革试点，规范行政事业性收费、价格调节基金、国有资本经营收益的管理，确保全面完成收入任务。

　　优化支出结构。按照保工资、保运转、保稳定、保重点的原则，统筹安排各项支出，加大教育、卫生、社会保障等方面的投入。全面深化预算改革，规范财政资金拨付制度，逐步将财政专项资金纳入财政集中支付范围。严格控制一般性经费支出，压缩机关公用经费开支，严禁新建办公楼，从严控制车辆购置，降低行政成本。加强政府债务管理，防范财政风险。

　　增强金融支持力度。搭建银企合作平台，建立互信共赢的银企合作关系。发挥市开发投资公司的作用，加强与开行陕西分行的合作，扩大政府信用贷款规模。鼓励银行开发新的金融产品，提高金融服务水平。加强金融生态环境建设，确保金融安全。

　　九、以关中城市群建设为契机，加快推进城镇化进程

　　城镇化是工业化发展的必然趋势，是加速推进工业化的重要载体。要抓住打造关中城市群的战略机遇，加快城市基础设施建设，完善城市服务功能，提升城市品位。

　　加强城市规划管理。按照关中城市群总体规划，统筹做好产业布局、交通网络、土地利用等规划衔接。强化规划先导作用，加大规划执法力度，维护城市规划的权威性。严格新区与耀州区城市规划的统一管理，从严审批建设项目。严格执行城乡建筑、工程设施抗震设防标准，做好项目的选址、设计和施工。

　　加快南市区开发建设。继续完善新区道路、供电、供排水、天然气等基础设施，完成纬九路、金达路建设工程，开工建设南部工业园区总出水口工程，绿化亮化鸿基路、铁诺南路、正大路等道路。铺设人行道2万平方米。实施建成区地下管网线系统建设。综合改造南市区中心广场。建成新耀垃圾无害化处理项目。加大耀州区旧城改造力度，完成华原路改造建设任务，实施沮河城市段绿化带工程，加快推进新耀一体化进程。

　　不断改善北市区人居环境。完成玉华路、河滨路和三里洞道路改造任务，整修红旗街至延安路、重兴路至河滨路道路。实施大同路立交桥维修和灯饰亮丽工程，完成一、二马路路灯改造。更新改造人行道10万平方米。建成2座人行天桥、3个街头广场，完成3公里漆水河河堤护栏建设任务。

　　进一步加大创卫工作力度。对照省级卫生城市标准，在继续加快硬件建设的同时，强化市容环境综合整治，着力提高市民素质。新建改造集贸市场26个，集中整治“八小行业”，坚决拆除城市主干道两侧的破旧建筑物和构筑物，加大对41个城中村的整治，改善城市环境面貌。建立长效机制，提高城市管理水平，力争创卫工作通过省上验收。

　　加快城镇建设步伐。加强村庄规划编制工作，加快列入“关中百镇”的9个城镇建设。深化户籍制度改革，推行城乡一体化户籍管理制度，促进人口有序流动。创新城镇建设投融资机制，吸引社会资本参与城镇建设。

　　十、以深化改革扩大开放为动力，增创科学发展新优势

　　要以纪念改革开放30周年为契机，以更大的决心推动改革，以更大的气魄扩大开放，增强经济发展活力。

　　全面提高资源配置市场化水平。对于自然性资源、公共性资源和资产性资源，凡可进行市场化运作的，一律实行有偿使用和公开转让。围绕推进基本公共服务均等化，改革财政资金投入方式和运行机制。充分发挥财政资金的杠杆作用，创新投资公司运行模式，健全风险分担机制和投融资协同机制，广泛吸引社会资本进入，构建以财政资金为引导的多元化投入格局。

　　继续深化以国企改革为重点的各项改革。完善国有资产监管体制，确保国有资产保值增值。大力推进秦岭水泥公司等国有企业战略性重组。积极稳妥地实施西北耐火材料厂、骊山水泥厂、市制药厂、市啤酒厂、市水泥厂和市变压器厂的政策性破产，完成市纺织厂公司制改造。加快推进国有商贸流通企业改革，做好国有粮食购销企业改革后续工作。加强与资产经营公司的协调，最大限度核销企业不良债务。在国企改革中，始终把保障职工合法权益放在首位，绝不能简单地走“一卖了之”、“关门走人”、买断工龄、把职工推向社会等老路子，而要创造更多的就业岗位，做到破产不破生产力、破产不破职工饭碗。继续深化财税、投资、盐业等项改革，加快事业单位分类改革步伐。

　　大力发展非公有制经济。坚持非禁即入，为非公有制经济创造宽松的发展环境。三年内逐步取消个体工商户工商管理费。市、区县都要设立中小企业发展专项资金，支持非公有制经济和中小企业发展。大力开展全民创业，鼓励党政机关工作人员离职、辞职创办企业；高校毕业生从事个体经营并符合规定条件的，三年内免交有关登记类、证照类和管理类收费；自主择业的军转干部、自谋职业的城镇退役士兵从事个体经营并符合条件的，三年内免征营业税、城市维护建设税、教育附加费和个人所得税。

　　努力扩大招商引资。把招商引资作为政府的中心工作，领导干部带头，群众广泛参与，大力推进全民招商。充分发挥企业招商引资的主体作用，引导企业拿出好的项目、优质资产开展招商。加大宣传推介力度，在招商重点区域，大力宣传我市的投资环境、产业优势、发展前景，增强客商投资愿望，提升铜川招商引资的吸引力。对招商引资项目，继续实行一个项目、一名领导、一套班子，强力推进，切实提高项目履约率和资金到位率。全年实际利用市域外资金10亿元以上。

　　推进科技创新和成果转化。落实企业创新优惠政策，引导企业加大投入，提升自主创新能力。加强与高等院校、科研院所的合作，加快耀州城郊型星火技术密集区、中星耐磨材料工程技术研究中心、印台小麦育种中心、耀州金圣生态农业示范基地等“13115”科技创新工程项目建设。加强人才队伍建设，大力培养企业家、高技能人才和应用开发型人才，积极引进人才，为经济发展提供人力支撑。继续实施农村基层人才队伍振兴计划，有效解决农村实用人才短缺问题。

　　十一、以实施“民生十大工程”为抓手，推进和谐社会建设

　　从今年开始，我市计划用五年时间，整体推进和实施教育工程、就业再就业工程、城乡居民增收工程、环境污染治理工程、社会保障工程、住房保障工程、采煤沉陷区综合治理和棚户区改造工程、农村基础设施建设工程、公共文化卫生体育建设工程、城乡社区服务体系建设工程等“民生十大工程”。这些工程，既有省上统一要求的，又有我市主动安排的。今年，本着需要与可能相统一、当前与长远相结合的原则，选择群众最迫切、经济社会关联度高的项目重点突破，让人民群众共享改革发展成果！

　　办好人民满意的教育。坚持教育优先方针，继续增加农村义务教育公用经费，适当提高农村家庭经济困难寄宿生的生活补助标准，从今年秋季起全面免除城市义务教育学杂费。做好贫困高中生、职中学生和大学生的资助工作，落实好国家生源地信用助学贷款政策，资助贫困高中生不少于1500人、职中学生不少于3500人、新录取的贫困家庭大学生不少于300人。完成农村27所学校2.8万平方米校舍改造任务。加快农村中小学布局调整，完善新区教育基础设施。加强教师队伍特别是农村教师队伍建设，培训教师3200人次。创建1个省级、2个市级教育强乡镇。巩固“普九”成果，王益区“双高普九”和宜君县义务教育均衡发展工作通过省政府评估验收，全市初中生辍学率控制在2%以内。大力发展职业教育，实施“人人技能工程”，建成2个省级职教示范骨干专业，启动建设省级综合性实训基地，完成职业高中招生4500人。

　　进一步健全公共卫生和医疗服务体系。整合卫生资源，提高服务水平。建设9所标准化社区卫生服务中心。完成乡镇卫生院基本医疗设备配置，建成200个标准化村卫生室。全市新型农村合作医疗参合率达到92%以上，将筹资标准由每人每年50元提高到90元。印台区计生优质服务先进区创建工作通过省上验收。

　　大力推进和谐文化建设。建成市图书馆主体工程。新建4个乡镇文化站和15个村文化室。全面启动数字电影工程，继续实施农村广播电视“村村通”工程，完成320个20户以上自然村“村村通”建设任务，新增农村有线电视用户5000户，新增数字电视用户2000户。强化文化市场管理，认真开展“扫黄打非”专项整治活动，净化文化传播渠道。开展奥运年全民健身运动，实施“农民体育健身工程”建设项目46个。本着热烈、隆重、节俭的原则，策划组织好建市50周年庆祝活动。

　　千方百计扩大就业。认真执行《就业促进法》，完善支持自主创业、自谋职业政策，加强就业和创业培训，使更多劳动者成为创业者，以创业促进就业。开展《劳动合同法》执法检查，切实保障劳动者的合法权益。大力扶持农民进城就业、失业人员再就业和大学毕业生就业，建立帮助零就业家庭解决就业困难的长效机制，帮扶3000名就业困难群体实现再就业。建设新区人力资源市场，完成职业技能培训1.4万人次。劳务输出5万人次。

　　努力增加城乡居民收入。加强企业职工工资分配调控和指导，严格执行最低工资制度。从今年起，再连续三年提高企业退休人员基本养老金水平。确保公务员津补贴和事业单位政策性增资按时足额兑现。落实带薪休假制度。多渠道增加农民收入，建立健全农民工工资支付保障机制，确保按时足额发放。鼓励群众通过诚实劳动与合法经营，普遍增加财产性收入。

　　不断完善社会保障体系。全面落实低保政策，确保城市低保对象人均月补差不低于80元，将年收入693元以下的农村贫困人口全部纳入低保范围。困难群体医疗救助实现全覆盖，将市属国有困难企业离退休人员纳入城镇职工基本医疗保险。启动城镇居民基本医疗保险试点工作，参保率达到50%。做好被征地农民社会保障、就业培训和新型农村社会养老保险试点工作。农村“五保”对象集中供养率提高20.7%。积极发展社会慈善事业。

　　改善城乡居民居住条件。加大沉陷区滑塌区居民整体搬迁和棚户区改造力度，加快经济适用房、廉租房建设，拆除危旧房8万平方米，完成红楼片区改造，启动青年路菜园公房棚户区改造。全年开工住房30万平方米，建成22万平方米，其中建成经济适用房12万平方米，开工建设廉租房3万平方米，安置困难群众2300户。强化住房公积金归集，市级财政全额供养人员公积金财政缴存比例由15%提高到20%。加快住宅小区集中供热步伐，规范物业管理，努力创造良好的居住环境。实施农民安居工程，解决1300名农民住房困难问题。

　　加强城乡社区服务体系建设。按照“四室三站一场”的标准，加快20个城市社区服务站建设，积极开展农村社区服务中心建设试点，推进城乡社会服务一体化、社区服务广普化。组织好第七次村民委员会换届选举，不断提高村民自治水平。

　　十二、以维护稳定为第一责任，创造良好的发展环境

　　坚持以人为本，紧紧依靠全市人民，最大限度地增加和谐因素，最大限度地消除不和谐因素，共同推进和谐社会建设。

　　切实加强社会管理。广泛开展全民信用道德教育，建立和完善社会征信体系。深入推进“五五普法”，提高全民法制意识。实施“十一五”应急体系建设规划，加强基层应急管理。加快网络与信息安全技术平台建设，强化网上虚拟社会管理。高度重视物价问题，从严控制涨价项目，确保粮油等基本生活必需品的市场供应，稳定居民消费价格水平。加强市场监管，严厉打击各种违法行为，努力维护市场秩序。

　　高度重视安全生产。始终坚持“安全第一、预防为主、综合治理”的方针，全面落实企业的主体责任和政府的监管责任，严格安全责任追究制度。深入开展煤矿、非煤矿山、交通运输、危险化学品、公共场所的安全专项整治活动，努力消除安全隐患，杜绝重特大事故发生。加强产品质量和食品药品安全监管，建成50个食品安全经营示范店，在10个主要农产品市场、超市实行农产品质量安全市场准入制度，让群众吃得放心、用得安心。

　　全力维护社会稳定。认真落实信访工作责任制、信访联席会议制度，健全群众权益维护机制，进一步加大矛盾纠纷排查工作力度，妥善处理群众的合理诉求和因征地拆迁、企业改制等引发的群体性事件。深入开展平安铜川创建活动，健全社会治安防控体系，加强城乡社区警务工作，全面完成公安“三基”工程建设任务，进一步提高群众对社会治安的满意率，实现“一控双降”目标。

　　十三、加强作风建设，提高领导和推动科学发展的能力

　　新形势、新任务对政府工作提出了更高的要求，要加快行政管理体制改革，切实转变政府职能，努力建设负责任、有作为、人民满意的政府。

　　转变政府职能。推进政企分开、政资分开、政事分开、政府与市场中介组织分开，减少政府对微观经济运行的干预。创新政府管理方式，寓管理于服务之中，更多地运用经济、法律手段调节经济活动。按照精简、统一、效能的原则，适当下移行政管理和行政执法重心。继续深化行政审批制度改革，进一步精简行政审批事项。加快电子政务建设，完善政务公开、新闻发布制度，保障人民群众的知情权、参与权、表达权和监督权。

　　推进依法行政。认真贯彻《国务院关于加强市县政府依法行政的决定》，完善重大行政决策的听取意见制度、听证制度、合法性审查制度、集体决定制度、实施情况后评价制度和责任追究制度等，切实将政府行为纳入依法管理轨道。健全行政监督制度，自觉接受人大监督、政协民主监督、社会舆论监督和人民群众监督，做到有权必有责、用权受监督、侵权要赔偿、违法要追究。

　　改进工作作风。大兴求真务实之风，倡导实干就是能力、落实就是水平的理念，坚持重实绩、说实话、办实事、求实效。切实精简会议、文件和检查评比。完善工作评价考核体系，强化数量、时间、标准和责任意识，以科学的机制引领发展方向，检验发展成果。认真执行《行政机关公务员处分条例》，推行行政问责制，奖优、治庸、罚劣。增强为民意识，做到重大决策依民而定，工作措施应民而出，安危冷暖唯民而系。

　　加强廉政建设。认真落实党风廉政建设责任制，坚持标本兼治、综合治理、惩防并举、注重预防的方针，完善教育、制度、监督并重的惩治和预防腐败体系。加强审计监督、财政监督和行政监察，完善工程招投标、经营性土地招拍挂、政府采购等制度，依法惩治商业贿赂行为，坚决纠正各类不正之风。政府工作人员要时刻牢记“两个务必”，艰苦奋斗，勤俭节约，坚决反对铺张浪费、大手大脚等奢侈之风，做到为民、务实、清廉。

　　各位代表，抢抓新机遇，实现新跨越，是时代赋予我们的光荣使命。让我们紧密团结在以胡锦涛同志为总书记的党中央周围，高举中国特色社会主义伟大旗帜，在市委的坚强领导下，同心同德，励精图治，为建设以现代建材业为主导的新型工业城市、实现率先发展跨越发展的目标而努力奋斗！
